# ﻿The genus *Platypalpus* Macquart (Diptera, Hybotidae, Tachydromiinae) from Inner Mongolia, China, with the description of four new species

**DOI:** 10.3897/zookeys.1198.114643

**Published:** 2024-04-23

**Authors:** Yuanyuan Wang, Ning Wang, Ding Yang, Kejian Lin

**Affiliations:** 1 Institute of Grassland Research, Chinese Academy of Agricultural Sciences, Hohhot, Inner Mongolia 010010, China Institute of Grassland Research, Chinese Academy of Agricultural Sciences Hohhot China; 2 Department of Entomology, College of Plant Protection, China Agricultural University, Beijing 100193, China China Agricultural University Beijing China

**Keywords:** Dance flies, Identification key, Inner Mongolia, new species, *Platypalpus*, taxonomy

## Abstract

*Platypalpus* Macquart is reported in Inner Mongolia, China for the first time. Four new species are found: *P.flavipilosus***sp. nov.**, *P.longus***sp. nov.**, *P.shengi***sp. nov.** and *P.shuimogouanus***sp. nov.** This paper provides a description of the four species and a key to the genus in Inner Mongolia.

## ﻿Introduction

*Platypalpus* Macquart, 1827 belongs to Tachydromiinae of Hybotidae. It is characterized by its raptorial mid leg and wing with an anal cell (Chvála 1975; Grootaert and Chvála 1992; Barták and Kubík 2015). The genus is cosmopolitan and found in all major zoogeographic regions, but over 75% of its 559 known species are from the Palearctic and Nearctic realms (Chvála and Wagner 1989; Grootaert 1992; Yang et al. 2007). Although most reports of the genus in the Palearctic region are from Europe, its presence in Asia is poorly documented (Barták and Shamshev 2015; Kanavalová et al. 2021). In China, 58 species have been reported, but none have been reported from Inner Mongolia, which borders Mongolia and Russia, despite the abundance of reports of the genus in these areas (Yang et al. 2007, 2018; Li et al. 2021).

**Figure 1.**
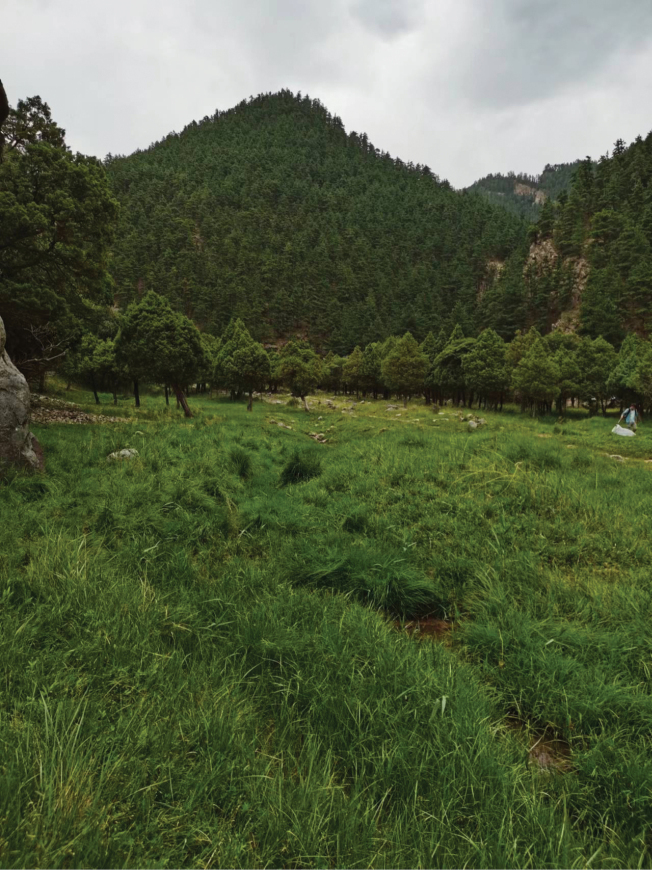
The habitat in Shuimogou.

Inner Mongolia is a long and narrow region located in the northern part of China, extending diagonally from northeast to southwest. The region is known for its low and uneven precipitation, strong winds, and significant seasonal variations, which nurture a diverse range of vegetation. Dongsheng District is situated in the southwestern part of Inner Mongolia and the eastern to central part of the Ordos Plateau. It has a temperate continental climate, which, coupled with its vast arid grassland, supports a unique ecosystem. Shuimogou is situated in the Helan Mountains of Inner Mongolia, a dry desert area in the middle temperate zone (Fig. 1). The region exhibits a clear vertical distribution pattern of climate, which is associated with various vegetation types, such as coniferous forests, broad-leaved forests, mixed coniferous and broad-leaved forests, scrub, grasslands, deserts, and meadows. This diverse vegetation is advantageous for the formation and maintenance of biodiversity.

In this research, the genus *Platypalpus* is newly reported from Inner Mongolia with four new species. A key to *Platypalpus* from Inner Mongolia is provided.

## ﻿Material and methods

The specimens used in this study were collected by sweep nets in Inner Mongolia in 2006 and 2010. All specimens are deposited in the Entomological Museum of China Agricultural University (CAU), Beijing. Morphological terminology follows Cumming and Wood (2009). The following abbreviations are used in the descriptions for the following bristles: **acr** – acrostichal bristles, **av** – anteroventral bristles; **dc** – dorsocentral bristles; **h** – humeral bristle; **npl** – notopleural bristles; **vt** – vertical bristles; presc – prescutellar bristles; **psa** – postalar bristles; **sc** – scutellar bristles.

## ﻿Taxonomy

### ﻿ Platypalpus

Taxon classificationAnimaliaDipteraHybotidae

Macquart, 1827

2B74F747-F917-544C-8F8D-16CF239D4721

#### Type species.

*Muscacursitans* Fabricius, 1775 (by designation of Westwood 1840).

#### Diagnosis.

Small to middle-sized, body length 2.0–4.0 mm; eyes narrowly separated on face; proboscis significantly shorter than head height, palpus very small, rounded; 2 pairs of vts; first flagellomere short-conical or long-conical; distinctive humerus, no distinctive humeral bristle; 2–6 rows of acrostichal bristles separated with dc; dense acrostichal on mesonotum, acr multiseriate, mixed with dc; costa vein terminating at apex of M_1+2_; subcostal vein not reaching costal margin of wings; R_4+5_ and M_1+2_ parallel or distinctly convergent apically; anal vein weak or absent; 1^st^ and 2^nd^ basal cell short; anal cell significantly smaller than basal cell; no discal cell; fore femur slightly thickened; mid leg raptorial, significantly thickened, with 2 rows of short black spine-like ventral bristle; mid femur slightly curved, with 1 sparse row of black spine-like ventral bristles and 1 apical spur, apex of male abdomen rotating to right (Chvála 1975; Grootaert and Chvála 1992).

### ﻿Key to species (males) of *Platypalpus* from Inner Mongolia

**Table:** 

1	Thorax wholly black	**2**
–	Thorax mostly dark yellow, somewhat blackish dorsally	***P.flavipilosus* sp. nov.**
2	First flagellomere, 1.9–2.0 times as long as wide; stylus, much longer than first flagellomere	**3**
–	First flagellomere, 4.9 times as long as wide; stylus, 1/3 as long as first flagellomere	***P.longus* sp. nov.**
3	One pair of vt; mid femur with brown anterior spot at tip; tarsomeres with narrow blackish apical annulation	***P.shengi* sp. nov.**
–	Two pairs of vt; mid femur without anterior spot at tip; tarsomeres without dark apical annulation	***P.shuimogouanus* sp. nov.**

### ﻿ Platypalpus flavipilosus sp. nov.

Taxon classificationAnimaliaDipteraHybotidae

139A80C2-3A21-550A-B332-B82CA49F1AF8

https://zoobank.org/8F176C04-4B67-4A62-A8D9-A8FADF7FB17C

Figs 2 , 6–9

#### Diagnosis.

Thorax dark yellow, somewhat blackish dorsally. Antenna yellowish; first flagellomere oval, short, slightly wider than pedicel, 1.3 times longer than wide. Mid tibia with 1 short finger-like apical spur.

#### Description.

**Male** (Fig. . 2). Body length 2.6–2.7 mm, wing length 2.7–2.8 mm.

***Head*** black, with pale gray pollen. Eyes narrowly separated on face; frons distinctly wider than face. Hairs on head yellowish, bristles black or brownish yellow. Ocellar tubercle with 2 oc and 2 short posterior hairs. 2 pairs of vt. Antenna (Fig. 6) yellowish; first flagellomere oval, short, 1.3 times longer than wide, slightly wider than pedicel, with indistinct blackish pubescence; stylus blackish, 2.9–3.0 times longer than first flagellomere, with short blackish pubescence. Proboscis yellowish, 0.6–0.7 times as long as head height. Palpus oval, longer than wide, yellowish, with yellow hairs and bristles.

**Figures 2–5.**
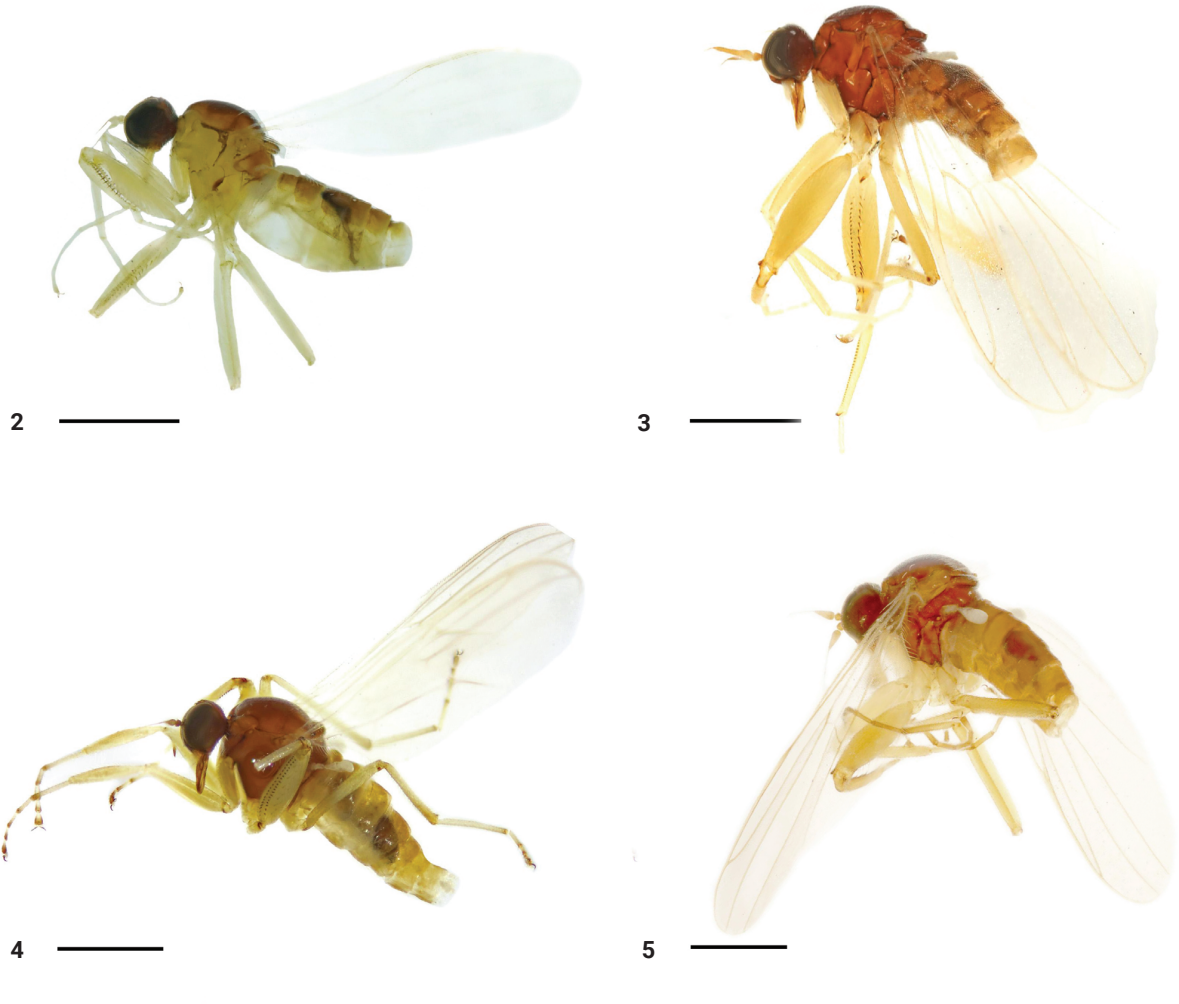
Habitus, lateral view **2***P.flavipilosus* sp. nov., male (dissected) **3***P.longus* sp. nov., male (dissected) **4***P.shengi* sp. nov., male (dissected) **5***P.shuimogouanus* sp. nov., male (dissected). Scale bars: 1 mm.

***Thorax*** dark yellow with thin pale gray pollen, blackish dorsally, humerus dark yellow, postalar callus brownish yellow; sutures of mesopleuron somewhat blackish. Hairs and bristles on thorax yellow, bristles strong; mesonotum with dense hairs, acr and dc multiseriate and not separated; 1 h, 2 npl (anterior bristle short), 1 psa, 1 presc; two pairs of sc (lateral pair short, 1/3 as long as apical pair).

***Legs*** yellow, but tarsomere V brown apically. Hairs on legs yellowish. Fore femur slightly thickened, 1.3–1.4 times wider than hind femur; mid femur distinctly thickened, 2.3–2.4 times wider than hind femur. Mid femur with 2 rows of short, black, spine-like, ventral bristles (few basal bristles brownish yellow, pv slightly longer than av), without row of outer pv. Mid tibia with 1 row of short black ventral bristles; apical spur short, finger-like (almost as long as tibia width). Wing hyaline, veins brownish; R_4+5_ and M nearly parallel, r–m and m–m contiguous. Squama yellow with pale hairs. Halter yellowish.

***Abdomen*** partly dark yellow, with pale gray pollen, sternites I–VI and hypopygium dark brown. Hairs and bristles on abdomen yellowish except those on hypopygium blackish. Hypopygium (Figs 7–9): left epandrial lamella short and small, apically widely obtuse. Right epandrial lamella extremely wide and large; right surstylus (Fig. 8) short and small, pointed at tip. Left cercus (Fig. 7) strongly dilated at base, narrowed towards apex, pointed at tip; right cercus almost as long as left cercus, narrow at tip, slightly curved inwards.

**Figures 6–9.**
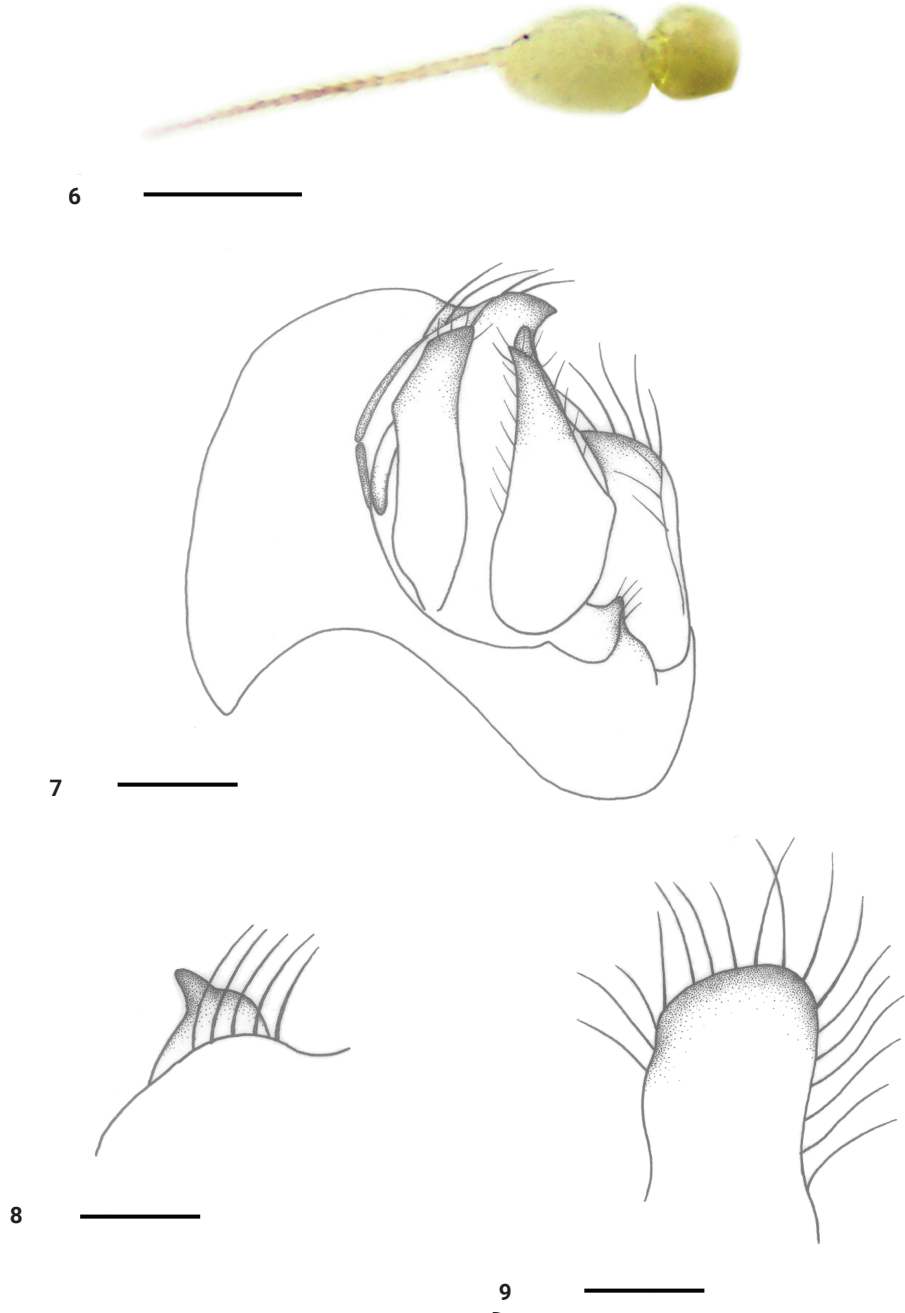
*Platypalpusflavipilosus* sp. nov., male **6** antenna **7** genitalia, dorsal view **8** right surstylus, lateral view **9** left epandrial lamella, lateral view. Scale bars: 0.2 mm.

**Female.** Body length 3.0–3.5 mm, wing length 2.9–3.0 mm. Similar to male, but abdomen dark brown except sternites I–IV or I–V dark yellow.

#### Type material.

***Holotype***: male, China, Inner Mongolia, Erdos, Dongsheng, 2006.VIII.7, Maoling Sheng (CAU). ***Paratypes***: 1 male, 6 females, same data as holotype (CAU).

#### Etymology.

The specific name refers to the yellow body hairs.

#### Remarks.

The new species belongs to the *P.longicornis* group. It is somewhat similar to *P.baotianmanensis* Yang, An et Gao from Henan of China, but can be easily distinguished from the latter by the wholly blackish mesonotum and postnotum. In *P.baotianmanensis*, the mesonotum is yellow with a wide black middle spot, and the postnotum is brownish-yellow laterally (Grootaert and Shamshev 2006).

### ﻿ Platypalpus longus sp. nov.

Taxon classificationAnimaliaDipteraHybotidae

D895B431-3374-5D8C-ADF3-58DCDD62E711

https://zoobank.org/B850E0AF-7F4A-49C8-9A7E-E7EBA445382C

Figs 3 , 10–13

#### Diagnosis.

One pair of vt. First flagellomere, 4.9 times longer than wide. Arista 1/3 as long as first flagellomere. Mid femur apically with 3–4 outer av. Mid tibia with short pointed, apical spur, almost as long as tibia width.

#### Description.

**Male** (Fig. 3). Body length 2.4–3.0 mm, wing length 3.3–3.4 mm.

**Figures 10–13.**
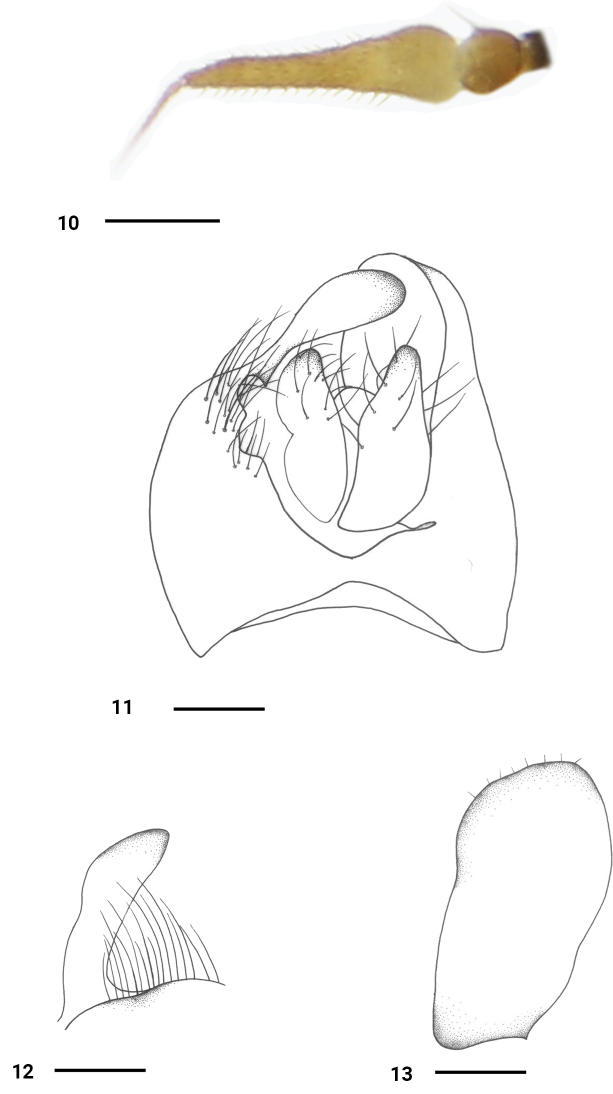
*Platypalpuslongus* sp. nov., male **10** antenna **11** genitalia, dorsal view **12** right surstylus, lateral view **13** left epandrial lamella, lateral view. Scale bars: 0.2 mm.

***Head*** black with pale gray pollen. Eyes narrowly separated on face; frons distinctly wider than face. Hairs on head yellowish, bristles brownish. Ocellar tubercle with 2 oc and 2 short hairs. 1pair of vt. Antenna (Fig. 10) blackish; first flagellomere very long, 4.9 times longer than wide, with short blackish pubescence; arista rather short, blackish, 1/3 as long as first flagellomere, with very short blackish pubescence. Proboscis dark brown, 0.7 times as long as head height; palpus yellowish, with yellow hairs and bristles, distinctly longer than wide, nearly acute at tip.

***Thorax*** black with thin pale gray pollen; mesonotum mostly subshiny. Sternopleuron with shiny spot. Hairs and bristles on thorax yellow; mesonotum with somewhat dense hairs, 6 rows of acr, narrowly separated with dc; 1 h, 2 long npl, 1 psa, 1 presc; 2 pairs of short sc (apical pair short, 1/3 as long as apical pair).

***Legs*** yellow, but tarsomeres V mostly dark brown with yellow base. Hairs and bristles on legs yellowish. Fore femur slightly thickened, 1.3 times wider than hind femur; mid femur distinctly thickened, 2.1–2.2 times wider than hind femur. Mid femur with 2 rows of short spine-like blackish ventral bristles (pv distinctly longer than av), 3–4 short spine-like outer av at tip, without row of outer pv. Mid tibia with row of black ventral bristles, apical spurt short, acute (nearly as long as tibia width).

***Wing*** hyaline, veins brownish; R_4+5_ and M nearly parallel; crossveins r–m and m–m slightly or distinctly separated. Squama yellow with pale hairs. Halter yellowish.

***Abdomen*** blackish with pale gray pollen. Hairs and bristles on abdomen yellowish. Hypopygium (Figs 11–13): left epandrial lamella (Fig. 13) rather narrow, apically widely obtuse. Right epandrial lamella wide; right surstylus (Fig. 12) rather long, finger-like. Left cercus (Fig. 11) slightly thickened at base, blunt and narrow at tip; right cercus slightly shorter, with shallow depression on outer edge of middle part, slightly widened at tip.

**Female.** Unknown.

#### Type material.

***Holotype***: male, China, Inner Mongolia, Erdos, Dongsheng, 2006. VIII.7, Maoling Sheng (CAU). Paratypes: 5 males, same data as holotype (CAU).

#### Etymology.

The specific name refers to the long right surstylus.

#### Remarks.

The new species belongs to *P.longicornis* group. It is somewhat similar to *P.longicornioides* Chvála, 1972 from Europe but can be distinguished from the latter by the 6 rows of irregular acr which are separated narrowly from the dc. In *P.longicornioides*, the acr bristles are biserial, and widely separated from the dc (Grootaert and Chvála 1992).

### ﻿ Platypalpus shengi sp. nov.

Taxon classificationAnimaliaDipteraHybotidae

E8F5435A-04C5-55A4-AC6E-17575555FCFE

https://zoobank.org/36E931EB-7107-435C-B9EB-6D31B24D914C

Figs 4 , 14–17

#### Diagnosis.

Mid femur with brown anterior spot at tip. All tarsomeres with blackish annulation at tip. Fore tibia distinctly thickened, with 3 spine-like dorsal bristles at middle and 2 rows of very long ventral hairs at apical 2/3.

#### Description.

**Male** (Fig. 4). Body length 3.1 mm, wing length 3.5 mm.

**Figures 14–17.**
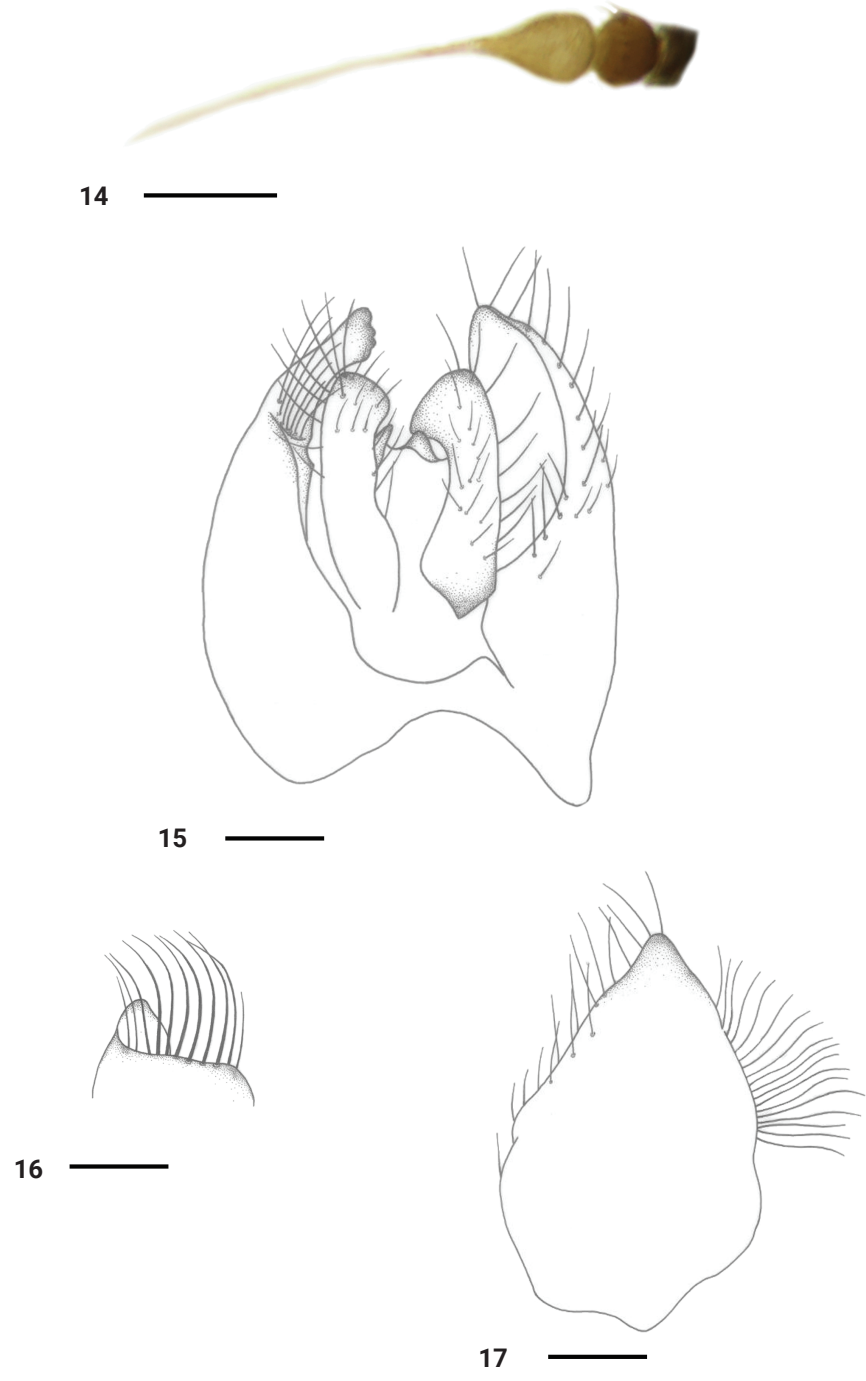
*Platypalpusshengi* sp. nov., male **14** antenna **15** genitalia, dorsal view **16** apex of right surstylus **17** left epandrial lamella, lateral view. Scale bars: 0.2 mm.

***Head*** black, with dense, pale gray pollen; clypeus pollinose. Eyes narrowly separated on face; frons slightly wider than face. Hairs and bristles on head yellowish. Ocellar tubercle with 2 oc and 2 short posterior hairs. 1 pair of vt. Antenna (Fig. 14) black, but first flagellomere blackish; first flagellomere short, subconical, with uniformly thin tip, 1.9 times longer than wide, with short brown pubescence; arista blackish, with short brown pubescence, 3.5 times longer than first flagellomere. Palpus dark yellow, short oval, with yellowish hairs and 2 short yellow bristles.

***Thorax*** black, with distinct pale gray pollen; mesopleuron with shiny black anterior spot. Hairs and bristles on thorax yellowish; hairs on mesonotum short and sparse; 1 weak h, 2 short npl, acr biserial, 1 long psa, 1 long presc; 2 pairs of sc (lateral pair short).

***Legs*** yellow, but tarsomeres with narrow, blackish, apical annulation; mid femur with brown anterior spot at apex. Hairs on legs mostly yellowish except tarsi with blackish hairs; bristles mostly black. Fore femur distinctly thickened, 1.4 times wider than hind femur; mid femur strongly thickened, 2.1 times wider than hind femur. Fore femur with 1 row of short yellowish av; mid femur with 2 rows of short, black, spine-like ventral bristles and 1 row of short outer yellow av and 1 row of long outer yellow pv; hind femur with 1 row of pale yellow av (apical av rather long). Fore tibia distinctly thickened, with 3 spine-like dorsal bristles at middle, 2 rows of very long ventral hairs at apical 2/3. Mid tibia with 1 row of black ventral bristles; apical spur long and acute, with 1 short terminal hair at tip.

***Wing*** hyaline, veins brownish yellow; cell R_4+5_ distinctly widened, M distinctly curved, R_4+5_ and M distinctly convergent apically, r–m and m–m distinctly separated. Squama yellow with pale hairs. Halter yellow.

***Abdomen*** subshiny brown, with pale gray pollen, but hypopygium blackish. Hairs and bristles on abdomen yellowish, but partly dark brown on hypopygium. Hypopygium (Figs 15–17): left epandrial lamella (Fig. 17) relatively narrow, longer than wide, apically slightly sharp. Right epandrial lamella relatively wide; right surstylus (Fig. 16) fused with right epandrial lamellas, basally short and wide, apically twisted and curved inwards, with row of long, marginal bristles. Right cercus (Fig. 17) almost as long as left cercus, weakly curved inwards apically, with weak bulge; left cercus apically widened and distinctly curved inward.

**Female.** Body length 3.0–3.5 mm, wing length 2.9–3.0 mm. Fore tibia slightly swollen, with 4 dorsal bristles.

#### Type material.

***Holotype***: male, China, Inner Mongolia, Erdos, Dongsheng, 2006. VIII.7, Maoling Sheng (CAU). ***Paratype***: 4 females, same data as holotype (CAU).

#### Etymology.

The specific name refers to the specimen collector, Professor Maoling Sheng.

#### Remarks.

The new species belongs to the *P.pallidiventris*-*cursitans* group. It is similar to *P.beijingensis* Yang et Yu from Beijing, but may be separated from the latter by the mid femur with a brown anterior spot at the tip, the distinctly thickened fore tibia with 3 spine-like dorsal bristles at middle and the 2 rows of long ventral hairs on the apical 2/3. In P.beijingensis, the mid femur is wholly brownish yellow, the fore tibia has no spine-like dorsal bristles and no long ventral hair (Yang and Yu 2005).

### ﻿ Platypalpus shuimogouanus sp. nov.

Taxon classificationAnimaliaDipteraHybotidae

D43760A3-040C-5AB0-87C5-2555B9482650

https://zoobank.org/AA797927-8E91-4A3D-AC9F-BD82A7603A8E

Figs 5 , 18–21

#### Diagnosis.

Two pairs of vt. Mid femur with 1 row of 3 long blackish outer pv on apical half. Mid tibia with 1 short, pointed, acute apical spur. First flagellomere 2.1 times longer than wide. R_4+5_ and M little convergent apically; crossveins r–m and m–m very narrowly separated.

#### Description.

**Male** (Fig. 5). Body length 2.7 mm, wing length 3.3 mm.

**Figures 18–21.**
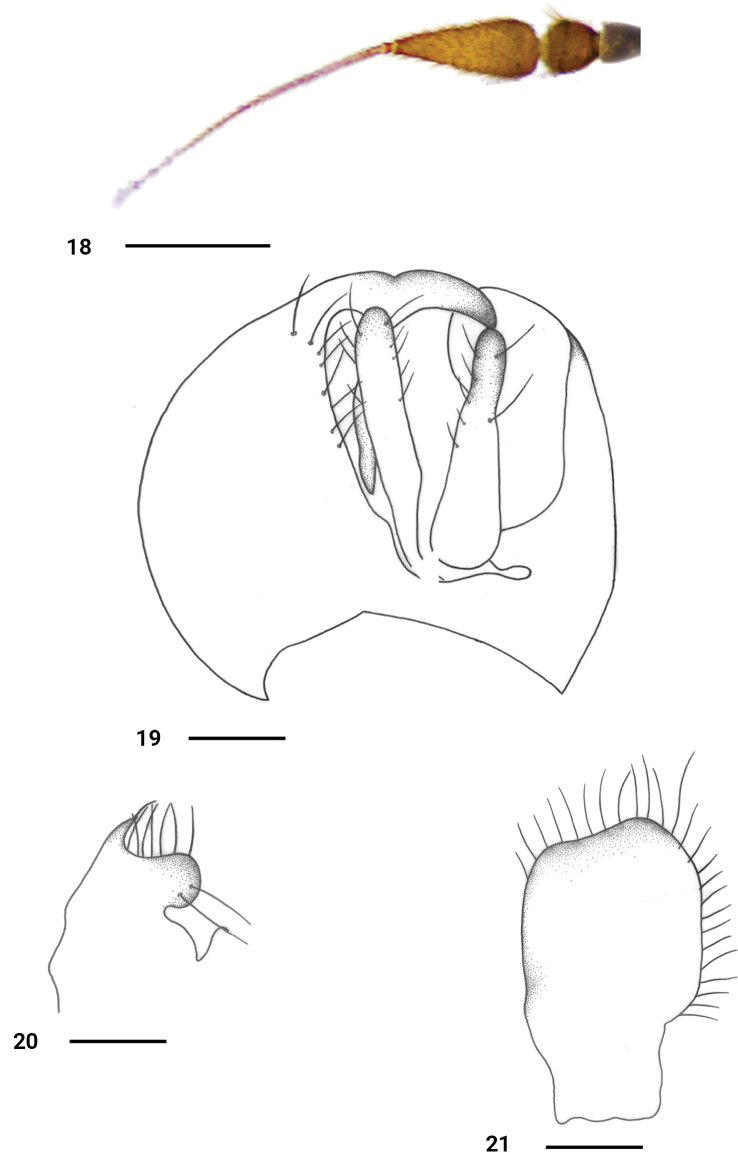
*P.shuimogouanus* sp. nov., male **18** antenna, lateral view **19** genitalia, dorsal **20** right surstylus **21** left epandrial lamella. Scale bars: 0.2 mm.

***Head*** black with distinct pale gray pollen; clypeus shiny black. Eyes narrowly separated on frons and face. Hairs yellowish on head, bristles blackish. Ocellar tubercle with 2 oc and 2 very short posterior hairs. 2 pairs of vt, outer vt short and curved inward. Antenna (Fig. 18) black; first flagellomere moderately long, subtriangular, 2.1 times longer than wide, distinctly blackish pubescent; arista black very long, 2 times longer than first flagellomere, with short blackish pubescence. Proboscis nearly blackish, 0.9 times as long as head height, palpus longer than wide, lobate, obtuse apically, yellowish, with 4 brown bristles (2 apical bristles long).

***Thorax*** black with thin pale grey pollen; mesonotum subshiny black with thin pollen; mesopleuron shiny black except postero-upper margin. Hairs on thorax yellowish, bristles yellow; mesonotum with dense hairs, acr and dc multiseriate and not separated; 1 h, 2 npl of subequal lengths, 1 psa, 1 presc; 2 pairs of sc (lateral pair short and weak, about 1/3 as long as apical pair).

***Legs*** yellow, but mid tibia brown, all tarsi brown to dark brown except tarsomere 1 brownish yellow with brown tip. Hairs on legs yellowish, but hairs on tibia and tarsi partly blackish. Fore femur weakly thickened, 1.2 times wider than hind femur; mid femur distinctly thickened, 1.5 times wider than hind femur. Fore femur with 1 row of pv (about 1/2 as long as femur width); mid femur with 2 rows of short spine-like black bristles (pv slightly longer than av) and 1 row of 3 long blackish outer pv on apical half. Mid tibia with 1 row of short, black, ventral bristles; apical spur short, pointed, shorter than tibia width.

***Wing*** hyaline, veins dark brown, R4+5 and M little convergent apically; 1^st^ basal cell slightly shorter than 2^nd^ basal cell, crossveins r–m and m–m very narrowly separated. Squama yellow with yellowish hairs. Halter yellowish.

***Abdomen*** black, with pale gray pollen. Hairs and bristles on abdomen yellowish. Hypopygium (Figs 19–21): left epandrial lamella wide, with wide and blunt apex. Right epandrial lamella very wide and large; right surstylus (Fig. 20) nearly fused with right epandrial lamella, somewhat narrowed, distinctly curved inward, with distinctly apical incision bearing several short marginal hairs. Left cercus (Fig. 19) nearly finger-like, slightly broader at base; right cercus almost as long as left cercus, finger-like.

**Female.** Unknown.

#### Type material.

***Holotype***: male, China, Inner Mongolia, Helan Mountain back, 2010.VIII.4, Yan Li (CAU).

#### Etymology.

The specific name refers to the type locality Shuimogou.

#### Remarks.

The new species belongs to the *P.pallidiventris*-*cursitans* group. It is somewhat similar to *P.henanensis* Saigusa et Gao from Henan of China (Saigusa and Yang 2002), but maybe separated from the latter by the yellow antenna and mid femur with 1 row of 3 long blackish outer pv on the apical half. In P.henanensis, the antenna is black, and the mid femur has 1 row of outer pv along the whole length (Yang and Yu 2005).

## ﻿Discussion

This study reports the first occurrence of *Platypalpus* Macquart, 1827 in Inner Mongolia. Four new species from Dongsheng and Shuimogou of Inner Mongolia are described. As the survey is restricted to only a part of the region, more reports of *Platypalpus* in Inner Mongolia are expected.

## Supplementary Material

XML Treatment for Platypalpus

XML Treatment for Platypalpus flavipilosus

XML Treatment for Platypalpus longus

XML Treatment for Platypalpus shengi

XML Treatment for Platypalpus shuimogouanus

## ﻿Additional information

### Author ORCIDs

Yuanyuan Wang https://orcid.org/0000-0003-1420-0567

### Data availability

All of the data that support the findings of this study are available in the main text.
